# A Rare Case of Anti-HMGCR and Anti-SRP-Positive Immune-Mediated Necrotizing Myopathy

**DOI:** 10.5339/qmj.2022.6

**Published:** 2022-02-22

**Authors:** Nariman Khan, Zehra Kazmi

**Affiliations:** ^1^UT Health San Antonio, Joe R. and Terry Lozano Long School of Medicine, Department of Internal Medicine, Texas, USA E-mail: Khan_nariman@yahoo.com; ^2^UT Health San Antonio, Joe R. and Terry Lozano Long School of Medicine, Department of Medicine, Division of Rheumatology, Texas, USA

**Keywords:** Anti-HMGCR, anti-SRP, immune-mediated necrotizing myopathy, statin use, rheumatology

## Abstract

Immune-mediated necrotizing myopathy (IMNM) or necrotizing autoimmune myopathy includes a set of distinct disorders associated with marked myasthenia, myofiber necrosis, and high creatine kinase levels. Anti-3-hydroxy-3-methylglutaryl coenzyme A reductase (anti-HMGCR) and anti-signal recognition particle (anti-SRP) are the two main autoantibodies associated with IMNM. Anti-HMGCR is usually associated with statin use. However, it may also be discovered in children without previous statin exposure, suggesting the existence of a complex genetic–environmental relationship in disease pathogenesis. Anti-SRP IMNM tends to present with more severe disease distinguished by pronounced myasthenia, worse neurologic outcomes, and treatment refractoriness. Its pathogenesis is also unknown; however, preliminary data suggest an antibody–complement-mediated mechanism of muscle cell lysis. Herein, we present the case of a 63-year-old man diagnosed with anti-HMGCR- and anti-SRP-positive IMNM that was treated with multiple immunosuppressants resulting in clinical improvement.

## Introduction

IMNM is an idiopathic inflammatory myopathy associated with myasthenia, myofiber necrosis, and elevated creatine kinase levels.^1^ IMNM has three main subtypes, namely, anti-signal recognition particle (SRP), anti-hydroxy-3-methylglutaryl coenzyme A reductase (HMGCR), and anti-HMGCR/SRP-negative disease.^2^ All subtypes are associated with significant morbidity and diminished overall well-being. However, data on disease pathogenesis and optimal treatment are limited. Herein, we highlight the rare case of a 63-year-old Hispanic man who was diagnosed with anti-HMGCR and anti-SRP-antibody-positive necrotizing autoimmune myopathy.

## Case Presentation

A 63-year-old non-smoking, non-alcoholic Hispanic man with partial trisomy 18, type II diabetes mellitus on metformin, hypertension, and coronary artery disease diagnosed in 2017 on aspirin, lisinopril, atorvastatin, and metoprolol for 3 years presented to clinic with a 3-month history of bilateral lower extremity soreness without preceding trauma. One month prior, the patient presented with similar complaints with elevated transaminases; subsequently, his general practitioner discontinued atorvastatin due to concern for statin-induced myopathy. On this visit, his labs were significant for further elevated aspartate aminotransferase to 171 U/L, alanine aminotransferase to 225 U/L, lactate dehydrogenase to 881 U/L, and creatine kinase to12,703 U/L. Gamma glutamyl transpeptidase, urine drug test, troponin, complete blood count, and viral hepatitis test were within normal limits. The patient was diagnosed with rhabdomyolysis and admitted to the hospital where he was started on intravenous normal saline. Magnetic resonance imaging of the left lower extremity revealed a tbl2 hyperintense signal in the adductors, hamstrings, and rectus femoris muscle consistent with myositis. Left anterior thigh biopsy revealed various stages of myofiber necrosis with myophagocytosis and regenerating myofibers related to IMNM. Prednisone 60 mg daily was initiated. Autoimmune workup revealed elevated anti-HMGCR and anti-SRP antibodies. Antinuclear antibody, erythrocyte sedimentation rate, and C-reactive protein were within normal ranges. The patient was eventually discharged with a 10-day course of steroids and instructed to avoid statins.

However, 2 weeks after finishing his prednisone-tapering schedule, the patient developed weakness of arms and calves along with dysphagia to solids. He was once again diagnosed with rhabdomyolysis (creatine kinase, 11,219 U/L) and treated with fluids and methylprednisolone 200 mg two times daily for 3 days, followed by prednisone 60 mg orally daily. He was also given rituximab 1000 mg inpatient, followed by another 1000-mg dose 2 weeks later with subjective improvement in strength. On follow-up clinical examination 2 weeks post-rituximab, he had 3/5 strength on hip flexion but could not lift his legs against gravity on straight-leg raise. He was also dependent on a walker to ambulate. The decision was then made to start the patient on intravenous immunoglobulin (IVIG) 2 g/kg for 5 days. He tolerated this therapy well and on repeat exam had improved strength, with 4/5 on lower limbs and 5/5 on upper limbs. Repeat laboratory exam revealed normal aspartate aminotransferase to 10 U/L, alanine aminotransferase to 23 U/L, and creatine kinase to 64 U/L.

## Discussion

Idiopathic inflammatory myopathies (IIMs) are immune disorders that primarily affect the muscular system. They include IMNM, inclusion body myositis, dermatomyositis, and polymyositis.^3^ Myopathies can be differentiated based on histopathologic findings on muscle biopsy. Our patient was diagnosed with IMNM, which has three subtypes including anti-SRP, anti-HMGCR, and anti-HMGCR/SRP negative.^2^ In general, all types of IMNM are associated with severe muscle weakness and myofiber necrosis with elevated creatine kinase levels.^1^ This case was rare because our patient was positive for both anti-HMGCR and anti-SRP antibodies, and data on this combination are currently limited.

IMNM induced by anti-HMGCR antibodies has an occurrence of 2–3 per 100,000 people who take statins, with higher risk in patients aged >50 years. This subtype can develop even after cessation of statins.^4^ The pathogenesis is unknown, but it is likely due to adverse interactions between genetics and environmental risk factors. It is hypothesized that statins induce overexpression of HMGCR in genetically susceptible individuals, particularly those with class II HLA-DRB1*11:01 allele, which may lead to the development of autoantibodies against HMGCR from the abnormal processing of this protein.^5^ In children, the HLA-DRB1*07:01 allele has a greater association with anti-HMGCR development, and most patients who develop the antibody have had no prior exposure to statins.^3^ This further suggests the presence of a complex genetic–environmental interaction. In patients who develop statin-induced IMNM, treatment includes permanent discontinuation of statins and immunosuppressive therapy individually or in combination with steroids, azathioprine, methotrexate, rituximab, mycophenolate mofetil, cyclosporine, cyclophosphamide, etanercept, or IVIG.^1^

Anti-SRP-induced IMNM tends to present with more intense proximal muscle weakness, greater elevation in creatine kinase, worse neurologic outcomes, and treatment refractory disease.^6^ This was evident in a study by Wantanbe et al. who examined 460 Japanese patients with IIMs. The authors discovered that compared with patients who had anti-HMGCR-related disease, patients with anti-SRP had more pronounced limb and neck myasthenia, muscle atrophy, dysphagia, respiratory distress, and required greater immunosuppression than those using steroids alone.^7^ The pathogenesis of anti-SRP necrotizing myopathy remains vague. However, in vitro experiments have shown that it may be secondary to an antibody–complement-mediated mechanism of muscle cell lysis.^8^ Benveniste et al. also found that anti-SRP levels correlated with creatine kinase quantities and muscle power in eight patients with anti-SRP-related necrotizing myopathy. This suggested that the antibodies themselves may be pathogenic.^9^

The treatment for anti-SRP-induced IMNM is similar to that of anti-HMGCR-induced disease, except that some experts recommend initial treatment with rituximab instead of other therapies because of better initial treatment response.^10^

Our patient possessed both anti-HMGCR and anti-SRP antibodies secondary to statin use and presented with significant myasthenia, dysphagia, and steroid-refractory disease. However, he responded well to both rituximab and IVIG treatments, indicating that both may be effective in patients with dual autoantibody-positive IMNM. This patient requires longer follow-up to monitor his response to these therapies. Thus far, the mechanism of how this patient possessed both autoantibodies rather than just one versus no autoantibodies remains unknown because of the rarity of this condition and the lack of documented similar cases.

## Conclusion

All subtypes of IMNM are associated with considerable morbidity and diminished well-being. Proper workup and aggressive treatment are necessary to prevent disease progression. More data on disease pathogenesis and optimal treatment of each subtype of IMNM are needed.

